# Paradigm Shift in Dendritic Cell-Based Immunotherapy: From *in vitro* Generated Monocyte-Derived DCs to Naturally Circulating DC Subsets

**DOI:** 10.3389/fimmu.2014.00165

**Published:** 2014-04-11

**Authors:** Florian Wimmers, Gerty Schreibelt, Annette E. Sköld, Carl G. Figdor, I. Jolanda M. De Vries

**Affiliations:** ^1^Department of Tumor Immunology, Radboud Institute for Molecular Life Sciences, Radboud University Medical Center, Nijmegen, Netherlands; ^2^Department of Medical Oncology, Radboud University Medical Center, Nijmegen, Netherlands

**Keywords:** dendritic cell vaccination, immunotherapy, naturally circulating dendritic cells, melanoma, monocyte-derived dendritic cells, plasmacytoid dendritic cells, myeloid dendritic cells

## Abstract

Dendritic cell (DC)-based immunotherapy employs the patients’ immune system to fight neoplastic lesions spread over the entire body. This makes it an important therapy option for patients suffering from metastatic melanoma, which is often resistant to chemotherapy. However, conventional cellular vaccination approaches, based on monocyte-derived DCs (moDCs), only achieved modest response rates despite continued optimization of various vaccination parameters. In addition, the generation of moDCs requires extensive *ex vivo* culturing conceivably hampering the immunogenicity of the vaccine. Recent studies, thus, focused on vaccines that make use of primary DCs. Though rare in the blood, these naturally circulating DCs can be readily isolated and activated thereby circumventing lengthy *ex vivo* culture periods. The first clinical trials not only showed increased survival rates but also the induction of diversified anti-cancer immune responses. Upcoming treatment paradigms aim to include several primary DC subsets in a single vaccine as pre-clinical studies identified synergistic effects between various antigen-presenting cells.

## Introduction

Melanoma is a malignant transformation of melanocytes – the pigment producing cells of the epidermis – and the most aggressive cancer of the skin (1). Over the past years, the number of melanoma incidences rose worldwide and reached 232,130 diagnosed cases in 2012 (2–4). Once melanoma patients develop metastatic disease, life expectancy drops and survival rates are low (1, 5, 6). Traditional treatment methods focus on chemotherapy and radiation therapy, which are highly invasive and often fail to induce objective clinical response (6).

Novel treatment strategies focus on melanoma patients that carry an activating mutation in protein kinases involved in MAPK or AKT signaling (7). Recently approved small molecule inhibitors, such as vemurafenib, allow specific targeting of these mutated kinases and lead to rapid tumor regression and prolonged survival in treated patients (7–9). However, due to the prompt development of resistance in many cases, and major cutaneous side effects, including the induction of neoplastic lesions, small molecule inhibitors are so far of limited clinical use (6, 8).

As pharmacological treatment paradigms fail to induce lasting responses, researchers, clinicians, and patients turn to immunotherapy, which – due to major advances – was recently declared as breakthrough of the year 2013 by scientific journal *Science* (10).

The ability of the immune system to fight tumors was first described by William B. Coley, who in the nineteenth century observed cancer regression in patients suffering from inoperable sarcoma after injecting bacterial toxins into neoplastic lesions (11). Today, cytotoxic CD8^+^ T lymphocytes (CTLs) are considered to be the fundamental mediators of anti-cancer immunity (12–16). *In vitro* experiments and studies in mice showed that CTLs are able to specifically target cancerous cells and destroy them by inducing apoptosis (12, 13, 17). Clinical evidence confirmed the importance of CTLs in patients suffering from melanoma and other cancers, as infiltrating CD8^+^ T cells found in tumor biopsies were strongly associated with improved life expectancy (18–20). Furthermore, melanoma patients with tumor-specific T cells in peripheral blood displayed increased clinical response rates (21). Immunotherapy hence aims to induce a potent and lasting T cell response against malignant cells.

One approach to potentiate the patient’s own immune response is to prolong the activity phase of the T cell response. Immunomodulatory drugs, such as the CTLA-4-blocking antibody ipilimumab or the PD-1-blocking antibody nivolumab, aim to unleash the patients’ natural anti-cancer T cell responses by interfering with inhibitory pathways (22–27). Neoplastic cells frequently exploit, e.g., the PD-1 pathways to suppress the immune system leading to immune escape and disease progression (28, 29). Notably, ipilimumab was the first treatment agent to provide survival benefit for patients suffering from melanoma and is now standard treatment for this type of cancer (10, 26, 28). Although only effective in a minority of patients, ipilimumab frequently induces objective responses that are remarkably long lasting (26, 30). Due to their broad mechanism of action, immunomodulatory antibodies can, however, cause severe and potentially fatal side effects by activating autoreactive T cells. Patients with, e.g., skin rash, colitis, hypophysitis, or high-grade hepatic adverse events were reported (6, 30). To overcome these side effects, targeted therapies that only activate cancer-specific T cells are desired.

Specific T cell responses are naturally induced by dendritic cells (DCs) (31, 32). DCs are professional antigen-presenting cells (APCs) that sample the body for antigens and danger signals derived from pathogens or tumors (33). After encountering such signals, DCs become activated and migrate to the lymph node, where they activate naïve T cells to become CTLs or helper T cells (32, 33). Due to their great regulatory capacities and outstanding ability to activate antigen-specific T cells, DCs have become an attractive target in several immunotherapeutic approaches in cancer.

**Figure 1:**
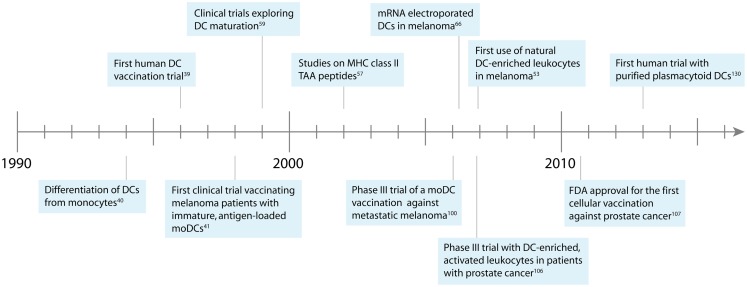
**Development of DC-based immunotherapy against melanoma**.

Cellular vaccination therapies were developed in the mid 1990s, when new laboratory techniques allowed the enrichment of DCs from peripheral blood (34–37). Murine DCs were isolated from peripheral blood by density gradients, loaded *ex vivo* with tumor antigens, and injected back into the blood (17, 38). This technique was rapidly transferred to the clinical setting when in 1996 pioneer Frank Hsu treated patients suffering from B-cell lymphoma with autologous, antigen-loaded DCs (39). Strikingly, clinical response could be detected in a majority of patients, kickstarting the field of therapeutic DC vaccination (Figure 1).

However, only after Sallusto and colleagues discovered a method to differentiate DCs from monocytes *in vitro*, sufficient cellular material was available to start clinical trials that went further than pure proof of principal (40). Following this development, Nestle and colleagues conducted the first DC vaccination trail in melanoma patients in 1998 (41). In this study, the group isolated autologous monocytes from peripheral blood of the patients and generated DCs *ex vivo*. Monocyte-derived DCs (moDCs) were subsequently pulsed with tumor-associated antigen (TAA) peptides or tumor lysate, and injected into the lymph nodes of the patients to activate the immune system. The results of this study were promising, as complete and partial responses could be observed in a number of patients. Furthermore, tumor-specific T cells were found in vaccinated patients, indicating the induction of a melanoma-specific immune response.

In the following years, a considerable number of phase I/II clinical trials explored the impact of various vaccination parameters on the treatment outcome. In this review, we will give an overview of the major advances in the field of therapeutic DC vaccination against melanoma since the initial study by Nestle. Further, we will highlight current developments focusing on natural DC subsets and their impact on immunotherapy, and we will conclude with an outlook on future vaccination strategies including the synergistic effects of DC subsets.

## Maturation of DCs

A major disadvantage of the DC vaccination protocol employed by Nestle et al. was the lack of activation signals. After differentiation, most moDCs possess an immature phenotype, which is dominated by high antigen uptake capabilities and poor T cell stimulatory abilities (42–45). Activation of DCs leads to the development of a mature phenotype characterized by upregulation of co-stimulatory molecules, major histocompatibility complex (MHC) molecules, and certain chemokine receptors (33, 46, 47). Especially the latter is of great importance for vaccination efficacy, as expression of the chemokine receptor CCR7 promotes the migration of injected DCs to the lymph nodes where the activation of T- and B-cells occurs (42, 47, 48). In addition to their inferior stimulatory capabilities, immature DCs were shown to induce antigen-specific tolerance, proposing that injection without activation signals is not only ineffective but also potentially detrimental (49).

While *in vivo* maturation signals primarily come from contact with pathogens or tissue injury, immature DCs can be matured by incubation with pathogen recognition receptor (PRR) agonists or cytokines such as TNF-α, and prostaglandin E_2_ (50, 51). In a clinical setting, CD40 ligation has also been used for DC activation (52, 53).

In 2003, a phase I/II clinical trial treating stage IV metastatic melanoma patients with autologous, antigen-loaded moDCs confirmed the superiority of mature DCs to induce strong immunity, as the immunological response against both included TAAs and the control antigen keyhole limpet hemocyanin (KLH) was improved in the majority of patients treated with mature DCs, as opposed to immature DCs (54). Strikingly, tumor regression could only be observed in patients of the mature DC arm, indicating that activating DCs prior injection improves clinical response as well. Other groups that employed modified maturation cocktails made the similar observations that DC maturation is necessary for the induction of a superior immune response (55–59). These results confirmed in a clinical setting what was already known for *in vitro* models: infused DCs need to express potent stimulatory molecules to generate a strong T cell response, especially when presenting cancer antigens with low immunogenicity. Nevertheless, as proper homing to the lymph nodes is a prerequisite for DC-mediated T cell activation, upregulation of CCR7 may also partly explain the observed differences (42).

## Route of Administration

In addition to maturation-induced upregulation of CCR7, the route of administration has a major impact on the migration of DCs to the T cell rich zones in the lymph nodes (42). Since intravenously (i.v.) injected, *ex vivo* generated DCs fail to induce potent skin-homing T cells in mice and appeared to be less efficient in inducing T_H_1 responses in humans, previous clinical trials focused on subcutaneous or intradermal (i.d.) administration of the vaccines (60–62). However, using ^111^In-labeling and scintigraphy, we could show that most of the injected DCs remain at the injection site, where they rapidly die to be phagocytosed by macrophages (42, 63, 64). Pretreatment of the skin with cytokines, toll-like receptor (TLR) ligands, or activated DCs did not lead to increased migration (64). Interestingly, Aarntzen et al. identified the number of injected DCs as an important factor for migration as a low cell density at the injection site correlated with high migration efficiency (64).

To further improve migration of DCs to lymph nodes and enhance the induced immune responses, different routes of administration have been explored in various studies (65, 66). Direct injection of DCs into the lymphatic system of the skin appeared to be a promising approach, as it ensures that most of the DCs reach the T- and B-cell rich zones of the lymph nodes. To test this hypothesis, our group conducted a phase I/II clinical trial and vaccinated melanoma patients with *ex vivo* generated, antigen-loaded, mature moDCs that were injected either intranodally or intradermally (65). Although intranodal vaccination led to increased DC migration to efferent lymph nodes, no difference in the frequency of tetramer-specific T cells could be detected. Furthermore, melanoma-specific T cells induced by i.d. vaccination turned out to be more functional, which might be caused by bystander activation of APCs at the injection site. Similar results have been found by Kyte et al. using mRNA transfected moDCs (66). Taking the complicated procedure of intranodal vaccination into account, intradermal injection of DCs appears to be the optimal route of administration in case of sufficient cellular material.

## T Cell Help

In the late 90s several groups independently discovered that, in absence of a strong inflammatory stimulus, DCs need to interact with CD4^+^ T cells to induce potent cytotoxic CD8^+^ T cells – a process called DC licensing (67–70). These findings, together with other important discoveries in the early 2000s, shifted the focus of therapeutic anti-cancer vaccination toward the CD4^+^ T cells and the impact of helper responses (71–73). Besides licensing DCs, T cell help plays a crucial role in memory generation and maintenance as well as affinity maturation of tumor-specific antibodies (72, 74, 75). Additionally, CD4^+^ T cells were shown to activate the innate immune system, to enhance the cytolytic function of macrophages, to induce senescence in malignant cells, and to destroy neoplastic cells directly (76, 77). The latter is of particular importance in the melanoma setting, where transformed melanocytes tend to constitutively express MHC class II molecules (78, 79). In particular, T_H_1 cells appear to be associated with favorable clinical outcome and overall survival (80). Despite this knowledge, integration of CD4^+^ T cell help in clinical trials was hampered due to the lack of defined TAA peptides binding to MHC class II molecules. To partly overcome this limitation, DCs were pulsed with unrelated antigens such as KLH or tetanus toxoid. The CD4^+^ T cells generated against these antigens were supposed to secrete interleukin (IL)-2 and pro-inflammatory cytokines, and to further activate the injected DCs, leading to an improved priming of cancer-specific CTLs (81). Whether or not the antigen-independent CD4^+^ T cell help had a strong effect on T cell priming could however not been definitely proven.

This changed when several groups characterized immunogenic melanoma-associated MHC class II epitopes of the tumor antigens gp100 and tyrosinase leading to a comparative study of melanoma patients treated with moDCs pulsed with both MHC class I and class II epitopes or MHC class I epitopes alone (79, 82–84). Analysis of patient samples showed that the simultaneous administration of TAAs restricted to both MHC classes lead to a broader anti-cancer T cell response with higher functionality compared to patients who received DCs loaded with epitopes for MHC class I only (79). Importantly, the tumor-specific CD4^+^ T cells were Foxp3 negative and displayed a T_H_1 phenotype, indicating that the vaccination did not induce regulatory T cells. This trend was reflected in the clinical response, as patients of the MHC class I/II arm showed increased progression free and overall survival, whereas no clinical benefit could be detected in patients of the MHC class I arm. The results thus indicate that antigen-specific CD4^+^ T cell help is indeed beneficial for the induction of a strong cancer-specific immune response, which is in line with a number of other studies (57, 85).

## Antigen Loading and Heteroclitic Peptides

Antigen loading was revolutionized when clinical grade mRNA electroporated moDCs became available. MRNAs coding for full-length TAA proteins containing multiple immunogenic epitopes were synthesized and used to transfect DCs (86, 87). In this approach, the transfected DCs translate the injected mRNA into full-length proteins, which are subsequently degraded by the proteasome and presented on MHC class I molecules (86). Adding an MHC class II targeting tag to the mRNA leads to the transport of translated proteins to exosomes and presentation on MHC class II molecules, necessary for priming CD4^+^ T cells (88, 89). Using electroporated DCs, several problems were solved: due to the presence of multiple immunogenic epitopes within the same protein, CD8^+^ and CD4^+^ T cells could be stimulated at the same time, and the induced immune responses became broader. The same effect rendered human leukocyte antigen (HLA)-restriction obsolete, as the various epitopes contained in each protein are able to bind to different HLA molecules. This made the enrollment of a much larger number of melanoma patients possible and increased the number of individuals potentially benefiting from this treatment (90, 91). These improvements however come with the price of reduced viability, which can turn into a serious problem when cellular material is scarce (92).

Studies using electroporated moDCs conducted by our group and others indeed showed the induction of specific CD4^+^ and CD8^+^ T cells in patients suffering from metastatic melanoma (63, 90, 91, 93). Interestingly, T cells specific for epitopes different from the TAA peptides employed in previous vaccines were readily detected in a number of patients, thus indicating an increased breadth of the immune response (93).

Soon after the first studies with electroporated moDCs were published, Bonehill et al. simplified the loading and activation process for moDCs distinctly. In their approach, they transfected DCs with mRNA, not only coding for TAA proteins, but also for the maturation-inducing molecules, CD40L and caTLR4 (constitutively active form of TLR4), as well as the T cell co-stimulatory molecule, CD70. This led to prolonged and enhanced maturation of DCs (90, 94, 95).

In parallel to the development of mRNA-based DC vaccines, various groups tried to improve the immunogenicity of the traditional peptide-pulsing approach to load DCs. Using rational design, researchers modified known TAA peptides by replacing single amino acids to improve binding to the MHC groove creating so called heteroclitic peptides (96–98). Due to tighter binding, heteroclitic peptides are presented for an extended time period, supposedly leading to stronger T cell activation. However, whereas many pre-clinical studies showed increased immunogenicity *in vitro*, clinical trials directly comparing modified and wild type peptides failed to measure any positive effect of heteroclitic peptides and even showed decreased frequencies of TAA-specific T cells in some patients (98). It appeared that the modified epitopes differed too much from the wild type peptide leading to the induction of T cells that were unable to detect endogenously presented antigens (99).

In summary, the development of mRNA electroporated moDCs simplified anti-cancer immunotherapy significantly as transfection of DCs not only induces a broad, HLA-independent CD4^+^ and CD8^+^ immune response but also reduces the time and costs for vaccine preparation. In contrast, heteroclitic peptides failed to prove superior immunogenicity in immunotherapy against melanoma.

## Efficacy of DC Immunotherapy

Although various vaccination parameters could be optimized and lasting responses were observed in selected patients, so far none of the conducted clinical trials using moDCs could demonstrate statistically supportable evidence for survival benefits in vaccinated patients. This became especially evident when in 2006 Schadendorf et al. published the first and so far only randomized phase III trial designed to demonstrate the clinical efficacy of moDC therapy in melanoma patients (100). The study was aborted early, as the Data Safety and Monitoring Board did not expect the group to reach the study goal. Analysis of the preliminary data could demonstrate the induction of an anti-cancer immune response in various patients but failed to show improved overall survival. Further, objective response was lower in the group of patients treated with DC vaccination as opposed to chemotherapy with dacarbazine (DTIC); thus no clinical benefit of moDC therapy could be detected.

One explanation for the observed lack of clinical response could be the inferior capacity of moDCs to induce effective anti-cancer immunity. However, as the study was already initiated in 1999 – thus only 1 year after the publication of the first phase I trial on moDC-based vaccines in melanoma by Nestle et al. – many of the aforementioned developments, including proper maturation of DCs, were not yet translated to the clinics (54, 100–103). Furthermore, several studies suggest that the employed maturation cocktail based on pro-inflammatory cytokines might not have been optimal for the induction of a strong anti-cancer immune response (51). DCs solely activated by these cytokines show only limited capabilities to produce polarizing cytokines that further decrease soon after activation – a phenomenon called exhaustion (51, 104, 105). At the time of injection, DCs thus might have possessed only limited capabilities to induce T_H_1 cells and CTLs. Additionally, the employed clinical protocols were not suited for multicenter trials leading to highly variable maturation levels and low numbers of generated DCs (100).

Interestingly, in the same year as Schadendorf et al. published their moDC study, Small et al. presented the results of a placebo-controlled phase III trial on DC-based immunotherapy in patients with metastatic asymptomatic hormone refractory prostate cancer (106). In contrast to Schadendorf et al., the authors employed a heterogeneous mixture of readily isolated leukocytes enriched for naturally circulating DCs by gradient centrifugation, thus avoiding long term *in vitro* culture. The leukocytes were activated and antigen-loaded using a recombinant fusion protein consisting of granulocyte-macrophage colony-stimulating factor and the TAA protein prostatic acid phosphatase. The prepared leukocytes were subsequently injected i.v. – <48 h after isolation. Strikingly, significantly increased overall survival and prolonged time to disease progression could be observed among patients of the treatment arm, thereby proving clinical efficacy of DC-based immunotherapy. Together with supporting studies, these results finally led to the first FDA approval for a cell-based therapy, Provenge^®^, in 2010 (107).

## Naturally Circulating DCs

Inspired by the promising results of the Provenge^®^ trial, we postulated that purified naturally circulating DCs would be superior in anti-cancer immunotherapy against melanoma (51). Not only are these DCs efficient in generating CTLs, they can also be readily isolated from the blood (108, 109). This allows immediate activation and antigen loading, thus avoiding long incubation periods and enabling robust standardization for use in multicenter trials. Therefore, natural DCs, despite their rare occurrence in peripheral blood, display various advantages over moDCs that are making them an attractive target for anti-cancer therapy.

**Figure 2:**
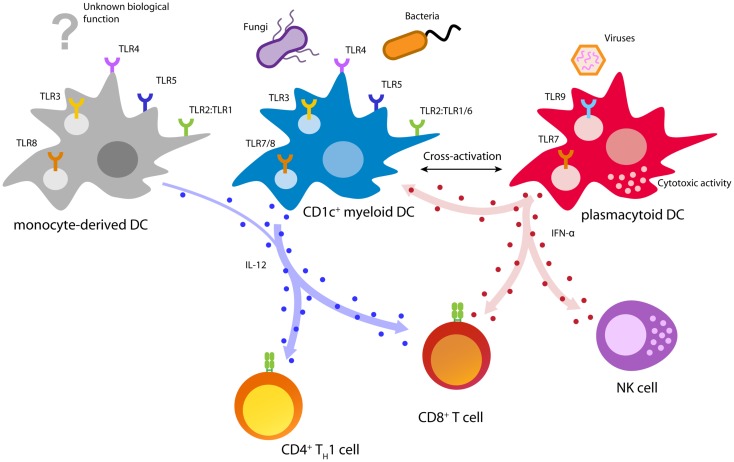
**Biology of immunotherapy-relevant human DC subsets**. Depicted are major DC functions relevant for pathogen recognition and DC activation, T cell priming, and anti-cancer immunity.

Human naturally circulating DCs can be divided into two main subsets: plasmacytoid DCs (pDCs) and myeloid DCs (mDCs), each with distinct phenotype and function during the immune response (Figure 2) (110). MDCs can be further subdivided in CD1c^+^ (BDCA1) DCs, CD141^+^ (BDCA3) DCs, and CD16^+^ cells, where the latter are considered to be more monocyte-like (111–115). MDCs are specialized in immunity against fungi and bacteria and have an enhanced ability to sense tissue injuries (110, 111). They are able to capture environmental- and cell-associated antigens and show high phagocytic activity (116).

CD141^+^ DCs are specialized in the detection and uptake of necrotic cells and excel in cross-presenting these antigens to T cells (117–120). Remarkably, CD141^+^ DCs uniquely express the C-type lectin CLEC9A (DNGR-1), which allows sensing of damaged cells by binding to exposed actin filaments (121, 122). In addition, CD141^+^ DCs can be activated using a distinct set of TLRs including TLR1, 2, 3, 6, and 8 (117, 123). Especially, TLR3 is strongly expressed and leads to upregulation of co-stimulatory molecules, as well as the secretion of pro-inflammatory cytokines and chemokines (117, 123). Upon activation, CD141^+^ DCs are able to secrete IFN-γ and IL-12, which allows the effective induction of T_H_1 and CTL responses (117, 119). However, due to the limited availability in blood and lack of GMP-grade isolation reagents, CD141^+^ DCs are currently not feasible for cellular immunotherapy. Several developments focusing on improved isolation and culturing, nevertheless, might allow their employment in future DC vaccination.

CD1c^+^ DCs are responsive to a great variety of microbial and fungal stimuli (124). Triggering of TLRs 1/2/6 by bacterial ligands leads to the activation of CD1c^+^ DCs and secretion of large amounts of the T_H_1-skewing cytokine IL-12 (123, 125, 126). Due to their potent antigen processing and presentation machinery, activated CD1c^+^ DCs are able to induce T_H_1 cells and cytotoxic T cells leading to a potent cellular immune response (108, 112, 117, 123, 126, 127). Moreover, *in vitro* studies showed that CD1c^+^ DCs isolated from healthy donors and prostate cancer patients are able to prime tumor-specific CD8^+^ T cells (108, 128).

In contrast to mDCs, pDCs are specialized in the detection and control of viral infections (110, 129). Viral infections are rapidly detected by pDCs via the engagement of TLR7 and/or TLR9 (116, 129). TLR triggering by viral agents leads to a rapid burst of type I interferons (IFNs) and induces cytotoxic functions in pDCs as well as natural killer (NK) cells (110, 130, 131). These outstanding antiviral activities make pDCs the key effector cells in early antiviral immunity (110). In a steady state, pDCs are characterized by low expression of MHC class II and co-stimulatory molecules (111). This phenotype is associated with tolerance induction and T_H_2 immunity, properties that are unfavorable for anti-cancer immunity (132). However, activation of pDCs leads to an upregulation of these proteins, turning pDCs into professional APCs that efficiently prime both, CD4^+^ and CD8^+^ T cells (108, 110, 131, 133). The strong release of type I IFN by pDCs leads to an IL-12 independent T_H_1 polarization characterized by strong secretion of IFN-γ and IL-10 (110, 134–136). Despite low antigen uptake and limited phagocytosis, pDCs isolated from blood, tonsils, and spleen were shown to efficiently cross-present antigens to CD8^+^ T cells (113, 120, 127, 137). Moreover, several studies reported that pDCs are able to prime potent melanoma-specific CD8^+^ T cells, which produce IFN-γ and are able to locate to melanoma lesions (108, 120, 138, 139). Finally, pre-clinical mouse models showed that pDCs are able to induce a tumor-specific T cell response *in vivo*, leading to control of tumor growth (138, 140).

## Naturally Circulating DC-Based Immunotherapy

Due to the low occurrence of naturally circulating DCs in blood, conclusive clinical evidence on their usability for immunotherapy is lacking. In 2006, a small-scale study by Davis et al. reported on a vaccine that employed Flt3 ligand (Flt3L)-mobilized naturally circulating DCs (53). The treatment was safe and strong immune responses were detected in several patients. However, the purity of the employed DCs was generally low and, as it turned out, the administration of Flt3L induced the expansion of regulatory T cells in melanoma patients (53, 141).

Encouraged by the promising pre-clinical data, we initiated the first clinical trial on a cellular vaccine based on purified pDC in 2008 at RadboudUMC in the Netherlands (142). PDCs were isolated from leukapheresis products using MACS separation kits and cultured overnight in IL-3. On the next morning, pDCs were activated with a conventional Frühsommer-meningoencephalitis (FSME; English: tick-borne encephalitis) vaccine, which has the benefit of sustained secretion of T cell stimulatory cytokines due to natural triggering of TLRs (143). Subsequently, pDCs were loaded with TAA peptides, and injected intranodally.

Initial tests revealed only mild side effects of pDC vaccinations and the toxicity was even lower as compared to moDC vaccinations (142). Further, pDCs were able to activate the innate immune system, indicated by a systemic type I IFN signature. PDCs were also shown to efficiently migrate to efferent lymph nodes and FSME-specific adaptive immune responses were detected in 14 of 15 enrolled patients. The potent stimulatory capacities of pDCs were reflected in the cancer-specific immune response, as 7 of 15 patients showed increased frequencies of gp100-specific T cells after vaccination. Strikingly, TAA-specific T cell clones with high avidity could be identified after vaccination, indicating the induction of a strong functional response. Nevertheless, the overall magnitude of the induced melanoma-specific immune response appeared to be limited compared to previous moDC vaccination trials, as the total frequency of specific T cells in blood of pDC-vaccine patients was rather low (65, 93). Further analysis of skin-infiltrating lymphocytes obtained from delayed-type hypersensitivity reactions against tumor antigens – a sensitive assay to analyze functionality, migration, and specificity of anti-cancer T cells – showed positive responses in only 2 out of 15 tested patients (142, 144). Despite this, the overall survival of patients treated with pDCs was greatly increased in comparison to matched controls treated with standard chemotherapy. However, assumptions on clinical efficacy have to be taken with caution, as the study was primarily designed to assess the safety and toxicity of pDC-based immunotherapy.

Nevertheless, the prominent survival benefit of vaccinated patients is especially interesting in respect to the low frequency of TAA-specific T cells. Two explanations for this phenomenon are likely: (I) T cells induced by pDCs might be more potent and functional as compared to moDC primed T cells. This could be due to different cytokine secretion patterns, differential expression of co-stimulatory molecules, improved migratory capacities, or prolonged survival. (II) Alternatively, instead of – or in addition to – inducing T cell responses, the focus of pDC-mediated anti-cancer immunity might lie on the activation of NK cells and the innate immune system. Evidence for this comes from the lasting type I IFN signature induced in vaccinated patients (142). Strikingly, various studies report on pDC-dependent, IFN-α-mediated activation of natural DC subsets in arteriosclerosis, autoimmunity, and infections (145–147). Furthermore, it could be shown that type I IFNs are able to activate NK cells, induce IFN-γ secretion, and enhance cytotoxicity (148, 149). However, in comparison to subjects that underwent recombinant IFN-α therapy, patients vaccinated with pDCs showed longer overall survival indicating that the observed clinical benefits were not induced by type I IFNs alone (150–152). Interestingly, it was shown that contact-dependent interactions between pDCs and lymph node DCs greatly enhance Ag presentation and priming of anti-herpes simplex virus CTLs (153). The authors identified CD2–CD2L and CD40–CD40L as key mediators of this effect. PDCs can thus activate other DC subsets, for instance mDCs, to potentiate the immune response. However, this synergy not only acts in one direction: mDCs were shown to mature pDCs and enhance their Ag presentation capabilities during bacterial exposure (116, 154). Interestingly, in one scenario pDCs only act as APCs without instructing T cells with polarizing cytokines (116). Together, these results show that natural DCs of various subsets cooperate with each other to enhance the immune response and that the roles in this regulatory network are variable and depending on the stimulus. However, the studies also indicate a hierarchical organization within natural DC synergies, with one DC subset orchestrating and polarizing the immune response, and the other merely acting as “zombie” APC without instructive capabilities (116).

Strikingly, mouse experiments demonstrated that injection of a mixture of *ex vivo* activated and antigen-loaded mDCs and pDCs induces a superior immune response against tumors (155). Moreover, therapeutic efficiency, as assessed by overall survival and tumor burden, was greatly improved when mice received simultaneous injections of both subsets compared to injections of one subset alone (155). The observed synergistic effect was mainly based on enhanced antigen presentation by mDCs induced by contact-dependent interactions with pDCs. These observations might explain why patients in our pDC vaccination trial showed significantly increased overall survival despite low frequencies of vaccination-specific CTLs (142, 155). Injected pDCs might have activated mDCs present at the site of injection leading to the induction of a T_H_1 and CTL response. As the *in situ* activated mDCs then would present naturally processed melanoma antigens expressed at the site of the tumor, the subsequently induced anti-cancer immune response would not be fully detectable when examining the vaccine-specific T cell response only.

Subsequent to the pDC-based vaccine, we conducted a phase I trial vaccinating metastatic melanoma patients with *ex vivo* activated and antigen-loaded autologous blood CD1c^+^ mDCs. Preliminary results confirm the safety and feasibility of mDC-based vaccines and could identify clinical responses in a number of patients (manuscript in preparation). Considering the results of these studies and the synergistic effects of pDCs and mDCs observed in mice and in *in vitro* models, the next step would be to initialize a human vaccination trial using a cocktail of activated and antigen-loaded mDCs and pDCs. Once injected in, e.g., the lymph node, these natural DC subsets might synergize and potentiate the T cell response.

Importantly, before clinical trials can exploit the synergy between mDCs and pDCs a number of questions need to be addressed: first: what ratio of mDCs and pDCs should be chosen and should one DC subset dominate the immune response? How should both DC subsets be activated *in vitro*? How does the simultaneous secretion of two different T cell polarizing cytokines (IFN-α by pDCs, IL-12 by mDCs) influence naive T cell priming? And what impact does this have on other immune cells? In addition, does the synergy between mDCs and pDCs also help to induce tumor-specific antibodies by B-cells? Does it increase the anti-cancer activity of the innate immune system?

*In vitro* studies and pre-clinical mouse models suggest answers to some of these questions. Mouse models, for instance, indicate that activated pDCs need to be cocultured with immature mDCs to induce maximal expression of IL-12 as well as co-stimulatory molecules CD40, CD80, and CD86 (Table 1) (155). This was cell–cell contact-dependent and also crucial for the induction of a superior CD8^+^ T cell response. Secretion of IFN-α by pDCs did not influence the secretion of IL-12 by mDCs, indicating that mDCs retain their strong T_H_1 polarizing capacities when administered together with pDCs. *In vitro* studies on human DCs, however, are not as conclusive and report on both, impaired and increased production of IL-12 by mDCs when cultured in IFN-α supplemented media (156–159). The induction of CD8^+^ T cells, however, seems to be augmented by the combined effect of IFN-α and IL-12 as comprehensive and lasting immune responses including effector and memory T cells could only be detected when T cells were cocultured with both cytokines (160, 161).

**Table 1:** **Controversial effect of IL-12 and IFN-α on immune activation and T cell priming**.

Species	Experimental setup	Observation	Reference
Mouse	Isolated pDCs were activated and cocultured with immature mDCs. This mixture or single DC subsets were then injected in tumor-bearing mice	The coculture of pDCs and mDCs induced strong expression of co-stimulatory molecules CD40, CD80, and CD86 on mDCs and led to superior secretion of IL-12 by mDCs. This process appeared to be contact-dependent. The induced T cell response was superior when both subsets were injected together and also led to improved tumor control	(155)
Human	Coculture of irradiated allogeneic moDCs and naive CD4^+^ T cells in αCD3-coated wells	Addition of type I IFNs to the cocultures led to decreased IL-12p40 production by DCs and the induction of IL-10 producing T cells	(156)
Human	PDCs and mDCs were isolated from blood and cocultured with cytokines. Subsequently, DCs were cultured with allogeneic, naive CD4^+^ T cells	IFN-α induced mDC maturation leading to IL-10 but not IL-12 production. IFN-α matured mDCs further induced IL-10 producing T cells	(157)
Human/mouse	MoDCs were activated in cytokine-supplemented media	The presence of type I IFNs at low levels augmented the production of IL-12p70	(158)
Human	MoDCs were activated using TLR ligands. IFN-α was added at different stages and secretion of IL-12 was measured	The presence of IFN-α during maturation increased the secretion of IL-12p70 by moDCs. When added after maturation IFN-α inhibited the secretion of IL-12p70	(159)
Human/mouse	Naive CD4^+^ T cells were activated in cytokine-supplemented media	In contrast to IL-12, IFN-α was not sufficient to induce stable T-bet expression and thus T_H_1 differentiation. However, no significant reduction in T_H_1 induction could be observed when both cytokines were administered together	(162)
Human	Naive CD8^+^ T cells were cultured and activated in αCD3/αCD28-coated plates. The media was supplemented with polarizing cytokines	Whereas IL-12 induced fast-dividing, IFN-γ secreting effector memory T cells, IFN-α primed slowly dividing central memory T cells. For a comprehensive T cell response, both cytokines were necessary	(161)
Human/mouse	Naive CD8^+^ T cells were cultured and activated via αCD3/αCD28-coated beats. The media was supplemented with polarizing cytokines	Priming of naive CD8^+^ T cells in IFN-α-supplemented media induced stem cell-like memory T cells with increased ability to respond to homeostatic cytokines, increased persistence upon adoptive transfer, and reduced effector functions. These T cells were able to mount robust recall responses and showed superior ability to contain tumor progression after adoptive transfer	(160)

Although many studies report synergistic effects of IFN-α and IL-12 on T cell priming and immune activation, it is hard to predict how these and other factors integrate in the complex microenvironment found in neoplastic lesions of melanoma patients. Following initial clinical trials focusing on safety and feasibility, future studies thus need to explore the interactions between DC subsets in patients and improve various vaccination parameters.

## Concluding Remarks

Although randomized clinical trials are needed to further prove the clinical efficacy of vaccination with natural blood DCs, DC therapy has major advantages over treatment with FDA-approved checkpoint inhibitors like ipilimumab, as DC therapy with natural DC is less costly and associated with only very mild side effects. Before anti-cancer therapy with natural DCs can be implemented as standard therapy for melanoma, some issues still need to be overcome. First, DC vaccination, in particular DC vaccination with natural DCs, is currently performed only in a limited number of medical centers. However, the isolation technique with magnetic beads is FDA-approved for stem cell isolation and common practice, thus enabling robust standardization for use in multiple centers in the future. In addition, as it is not feasible yet to perform mRNA electroporation on these rare cells, antigen loading still depends on HLA-binding tumor-peptides, thus excluding patients that do not have the matching HLA-phenotype. Efforts are made to enable peptide-loading for a broader HLA-repertoire, including MHC class II epitopes, to induce broader immune responses and enable inclusion of more patients.

As the field of moDC vaccinations appears to have reached some level of maturity, naturally circulating DC-based vaccinations are just at the beginning of their clinical development. However, the lessons learned from moDC-based vaccination trials will surely contribute to accelerate the development of mDC/pDC-based vaccines, hopefully leading to highly efficient DC-based immunotherapies and benefits for an increasing number of cancer patients.

## Conflict of Interest Statement

The authors declare that the research was conducted in the absence of any commercial or financial relationships that could be construed as a potential conflict of interest.

## Abbreviations

APC, antigen-presenting cell; CTL, cytotoxic T lymphocyte; DC, dendritic cell; FSME, Frühsommer-meningoencephalitis; HLA, human leukocyte antigen; i.d., intradermal; IFN, interferon; IL, interleukin; i.v., intravenous; KLH, keyhole limpet hemocyanin; mDC, myeloid dendritic cell; MHC, major histocompatibility complex; moDC, monocyte-derived dendritic cell; NK cell, natural killer cell; pDC, plasmacytoid dendritic cell; PRR, pathogen recognition receptor; TAA, tumor-associated antigen; TLR, toll-like receptor.
